# Diagnostic Pitfalls of CT in Malignant Superior Cerebellar Artery Infarction: Implications for Treatment Decisions and Future Management Strategies

**DOI:** 10.3390/jcm14228229

**Published:** 2025-11-20

**Authors:** Maria Gollwitzer, Baran Atli, Vanessa Seiter, Tobias Rossmann, Eva Horner, Anna Hauser, Gracija Sardi, Verena Sölva, Andreas Gruber, Kathrin Aufschnaiter-Hiessböck

**Affiliations:** 1 Department of Neurosurgery, Kepler University Hospital, Johannes Kepler University Linz, 4020 Linz, Austria; 2 Medical Faculty, Johannes Kepler University Linz, 4020 Linz, Austria; 3 Clinical Research Institute for Neuroscience, Johannes Kepler University Linz, 4020 Linz, Austria

**Keywords:** stroke, posterior fossa stroke, CT, MRI, decompressive craniectomy, prognostic imaging, PRISMA

## Abstract

**Background/Objectives:** Superior cerebellar artery (SCA) infarction is a rare but clinically significant subtype of posterior circulation stroke. Extensive swelling in the SCA territory may cause downward brainstem compression and appear as brainstem hypodensity on computed tomography, potentially leading to premature treatment withdrawal. **Methods:** We report the case of a 50-year-old woman with acute SCA-territory infarction (NIHSS = 7) presenting with vertigo, dysphagia, dysarthria, and diplopia. Initial computed tomography suggested extensive brainstem infarction, prompting withdrawal of treatment. Diffusion-weighted MRI revealed reversible edema with brainstem sparing. The patient underwent suboccipital decompressive craniectomy and ventricular drainage with favorable neurological recovery. In addition, a systematic literature search was conducted according to PRISMA 2020 guidelines in PubMed, Web of Science, and Scopus (studies published since 1 January 2015). Fifteen studies met predefined eligibility criteria. **Results:** Magnetic resonance imaging findings were decisive in avoiding a falsely dismal prognosis and inappropriate withdrawal of care. Across the literature, infarct volume (>30–35 mL), brainstem involvement and bilateral cerebellar infarction emerged as key predictors of malignant course. Early decompressive surgery was consistently associated with improved survival, though functional outcomes varied. Fast magnetic resonance imaging techniques and volumetric imaging improved risk stratification and surgical decision-making. **Conclusions:** SCA infarction can mimic brainstem infarction on computed tomography due to secondary compression rather than true ischemia. Magnetic resonance imaging is essential to guide treatment and prevent avoidable mortality. Multimodal imaging combined with interdisciplinary management allows for accurate prognostication and optimized surgical timing in malignant SCA infarction.

## 1. Introduction

Superior cerebellar artery (SCA) infarction represents a rare but clinically relevant subtype of posterior circulation stroke, accounting for less than 2% of all ischemic strokes and approximately 15% of cerebellar infarctions [1,2]. The SCA supplies the superior cerebellar hemispheres, the vermis and parts of the rostral brainstem and midbrain. Infarction in this territory typically presents with dizziness, ataxia, dysarthria, diplopia, skew deviation or sensory disturbances, but often spares the lower brainstem and cranial nerve nuclei, making early diagnosis challenging [1,3]. Because these symptoms may overlap with benign vestibular disorders and often lack focal neurological deficits, SCA infarction is frequently underdiagnosed in the hyperacute stage.

A critical complication of cerebellar stroke is malignant cerebellar edema, which may develop in 17–54% of cases and is associated with mortality rates of up to 80% without surgical intervention [4]. In SCA infarction, swelling often causes downward compression of the brainstem rather than direct parenchymal involvement. This can mimic brainstem infarction on early computed tomography (CT) scans, potentially leading to premature treatment withdrawal [3,5,6]. Differentiating true ischemia from reversible edema is therefore crucial for timely and appropriate therapeutic decisions. This diagnostic pitfall is particularly impactful in SCA infarction, where swelling patterns differ anatomically from PICA or AICA strokes and may produce misleading radiological appearances despite initially preserved brainstem function.

CT remains the most widely used initial imaging modality in acute stroke due to its availability and speed. However, its sensitivity in the posterior fossa is limited because of beam-hardening artifacts and the compact anatomy of this region [5,6]. Hypodensity in the brainstem may reflect either true infarction or secondary mass effect from cerebellar swelling [6]. MRI, particularly diffusion-weighted imaging (DWI) and apparent diffusion coefficient (ADC) mapping, offers higher sensitivity for detecting infratentorial ischemia and can distinguish between cytotoxic edema and infarction [5]. Early MRI evaluation therefore plays a pivotal role in accurately confirming the diagnosis, avoiding false-negative prognostication and guiding surgical decision-making in patients with suspected malignant cerebellar infarction.

Several clinical and radiological markers have been proposed to predict malignant course and poor outcome in cerebellar infarction. Volumetric thresholds, such as an infarct volume exceeding 25–30 mL or an infarct-to-posterior-fossa ratio > 0.25, have been associated with neurological deterioration and the need for early surgical intervention [7,8,9]. Additional predictors include bilateral lesions, brainstem compression, and reduced level of consciousness at presentation [8,9,10]. However, most available studies pool SCA, PICA, and AICA infarctions, despite their distinct vascular territories and mass-effect dynamics. As a result, prognostic thresholds and imaging findings may not accurately reflect the specific course of isolated SCA-territory strokes.

Despite this existing knowledge, specific evidence for SCA-territory infarctions remains scarce, as most studies combine SCA with Posterior inferior cerebellar artery (PICA) and Anterior inferior cerebellar artery (AICA) infarctions. Given the unique topographic and radiological characteristics of SCA stroke, further synthesis of current evidence is warranted. This review therefore aims to:

(1) Illustrate a representative clinical case of malignant SCA infarction.
(2) Provide a structured synthesis of the literature published since 2015.
(3) Discuss diagnostic and therapeutic implications with a particular focus on imaging-based decision-making. By integrating clinical, radiological, and surgical perspectives, this work aims to clarify diagnostic pitfalls and highlight evidence-based strategies for improving outcomes in malignant SCA infarction.

## 2. Materials and Methods

### 2.1. Case Documentation

Clinical, radiological and therapeutic data of a patient with acute ischemic stroke in the SCA territory were retrospectively analyzed. Neuroimaging included non-contrast cranial CT, MRI sequences—DWI, ADC, fluid-attenuated inversion recovery (FLAIR), and time-of-flight angiography (TOF)—as well as digital subtraction angiography (DSA). The diagnostic process, therapeutic decisions, and clinical course were thoroughly documented and jointly reviewed by a neurologist, neuroradiologist and neurosurgeons. Written informed consent for publication was obtained from the patient’s next of kin. Approval by the local ethics committee was waived for publication of a case report.

### 2.2. Literature Review Methodology

This review was conducted in accordance with PRISMA 2020 guidelines. The study was not prospectively registered, as registration is not mandatory for narrative systemic reviews. Reporting, screening and synthesis procedures followed PRISMA principles for transparency. A completed PRISMA 2020 checklist is provided in the Supplementary Materials.

#### 2.2.1. Search Strategy

A systematic search of PubMed, Scopus, and Web of Science was performed for publications from 1 January 2015 to 31 August 2025. The search targeted studies reporting imaging findings, prognostic markers or therapeutic management in space-occupying cerebellar infarction, including isolated SCA-territory infarctions and mixed cerebellar stroke cohorts.

The search strategy combined controlled vocabulary and free-text terms. An example PubMed search string was:

“superior cerebellar artery infarction” OR “SCA stroke” OR “cerebellar infarction” OR “posterior fossa infarction” AND

“malignant cerebellar infarction” OR “decompressive craniectomy” OR “ventricular drainage” OR “computed tomography” OR “magnetic resonance imaging”.

Equivalent adaptations were used for Scopus and Web of Science. All results were imported into a reference manager, where duplicates were removed.

#### 2.2.2. Study Selection

Two reviewers independently screened titles and abstracts, followed by full-text assessment of all potentially eligible records. Discrepancies were resolved by consensus.

Inclusion criteria were:

(1) adult patients with cerebellar infarction involving the SCA territory,
(2) availability of imaging and/or clinical data relevant to diagnosis, prognosis, or surgical management, and
(3) original clinical or radiological data.

Exclusion criteria included non–space-occupying infarctions, pediatric populations, experimental or animal studies and single case reports lacking clinically relevant outcome data.

### 2.3. Data Extraction and Synthesis

Data extraction was performed using a structured template capturing authorship, publication year, study design, sample size, imaging modality, infarct territory, volumetric measures, prognostic variables, treatment strategies and clinical outcomes.

Because of heterogeneity in study design, imaging thresholds and outcome measures, meta-analysis was not feasible. A structured qualitative synthesis was performed across four predefined thematic domains:

1. Imaging characteristics, including CT–MRI comparisons, volumetric assessments and posterior fossa ratios;
2. Prognostic markers, such as infarct volume, brainstem involvement and bilateral cerebellar lesions;
3. Therapeutic strategies, covering timing of intervention, decompressive surgery, ventricular drainage and minimally invasive techniques;
4. Functional outcomes, including reported recovery patterns and long-term neurological status.

This thematic approach allowed a clinically oriented synthesis of diagnostic and prognostic elements essential for treatment decision-making in malignant SCA infarction.

## 3. Results

### 3.1. Illustrative Case

**Figure 1:**
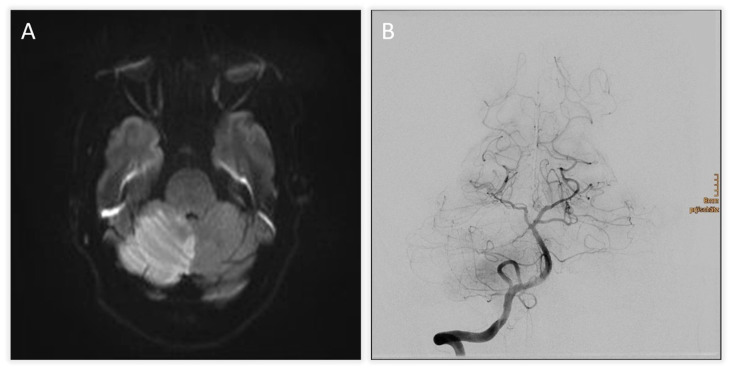
(**A**) Diffusion-weighted MRI on admission showing acute ischemic infarction of the right cerebellar hemisphere and vermis. (**B**) DSA demonstrating occlusion of the right SCA with preserved patency of the basilar tip.

A 50-year-old woman with no prior medical history presented with acute severe vertigo, dysphagia, dysarthria, and diplopia. Neurological examination revealed skew deviation, left-sided hemihypesthesia, bilateral bradykinesia, and ataxia (NIHSS score 7). Initial diffusion-weighted MRI showed acute ischemia of the right cerebellum and vermis (Figure 1A).

**Figure 2:**
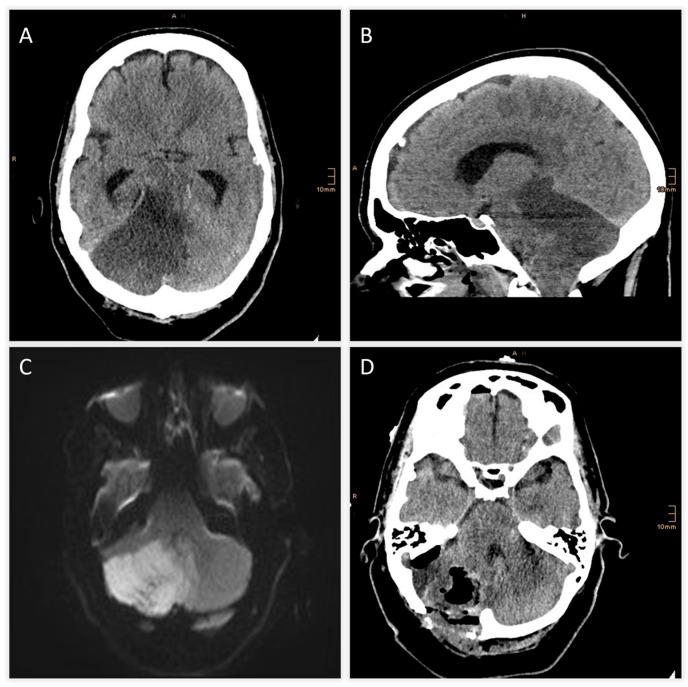
(**A**,**B**) Follow-up CT revealing an expanding infarct of the right cerebellum with mass effect and apparent hypodensity in the pons, midbrain and diencephalon, raising suspicion of brainstem infarction. (**C**) Axial diffusion weighted image obtained after suspicious CT findings excluding brainstem infarction and demonstrating predominantly cerebellar edema. (**D**) Postoperative CT scan following suboccipital decompression, partial cerebellar tissue resection and ventricular drainage.

TOF-angiography identified a thrombus at the proximal SCA. Intravenous thrombolysis was administered, followed by DSA confirming right SCA occlusion with preserved basilar patency (Figure 1B). Mechanical thrombectomy was attempted but unsuccessful. Follow-up CT revealed extensive hypodensity in the right cerebellum with mass effect and apparent extension into the brainstem, raising concern for infarction and poor prognosis (Figure 2A,B). Immediate MRI, however, excluded brainstem infarction and confirmed predominantly reversible edema (Figure 2C). MRI demonstrated vasogenic edema rather than cytotoxic infarction, characterized by T2/FLAIR hyperintensity with preserved ADC values and only minimal diffusion restriction. This pattern indicated reversible swelling, in contrast to the marked ADC decrease expected in true parenchymal infarction Emergency suboccipital decompression, partial resection of infracted parenchyma and ventricular drainage were performed (Figure 2D). Following suboccipital craniectomy, the cerebellar surface remained markedly swollen and non-pulsatile. A small corticotomy was performed, and non-viable infarcted tissue was gently removed to achieve adequate decompression. Finally, an external ventricular drain was placed to manage hydrocephalus.

The patient was extubated after 10 days. Further etiological work-up revealed a patent foramen ovale with atrial septal aneurysm, which was treated by interventional cardiology. A detailed timeline of clinical events and interventions is provided in Table 1.

### 3.2. Review of the Literature

Initial literature screening yielded 386 records. After removal of 97 duplicates, 289 studies remained for title and abstract screening. A total of 270 studies were excluded because they did not meet the predefined eligibility criteria.

Nineteen full-text articles were retrieved for detailed assessment. Four were excluded due to insufficient imaging or clinical data relevant to the study objectives.

**Figure 3:**
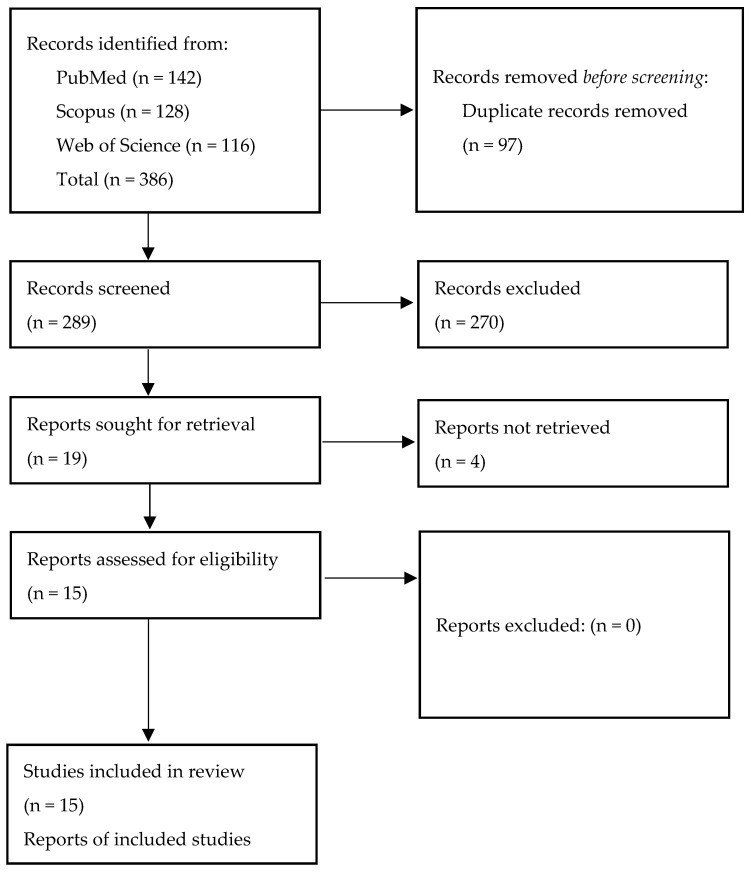
PRISMA flow diagram illustrating the study selection process for the structured literature review on malignant SCA infarction.

Ultimately, 15 studies met all inclusion criteria and were included in the qualitative synthesis (Figure 3).

#### 3.2.1. Study Characteristics

A total of fifteen studies published between 2016 and 2025 met the predefined inclusion criteria and were included in the qualitative synthesis. Thirteen were retrospective observational studies, three of which were multicenter in design. Two additional systematic reviews were incorporated. Together, these studies provided a comprehensive overview of the diagnostic, prognostic and therapeutic aspects of space-occupying cerebellar infarctions involving the SCA territory.

#### 3.2.2. Diagnostic Imaging

Initial brain CT is often performed in patients with suspected SCA infarction; however, CT has limited sensitivity in detecting posterior fossa strokes, particularly in the brainstem region. Several studies emphasized the diagnostic value of MRI, especially diffusion-weighted imaging, for confirming cerebellar infarction and identifying brainstem involvement when CT findings are inconclusive. Kim et al. (2016) reported that MRI was crucial for detecting brainstem infarcts in cases where CT findings did not align with clinical symptoms [10]. Similarly, Kapapa et al. (2024) found that although 60% of patients underwent CT initially [7], MRI was required in 40% to establish the diagnosis. MRI consistently revealed the true extent of ischemia in the SCA territory, which CT often underestimated. These findings underscore the importance of early MRI in guiding management when CT results are equivocal or show only subtle changes.

#### 3.2.3. Prognostic Factors

Infarct volume emerged as the most consistent and clinically relevant prognostic marker across nearly all studies. Baki et al. (2025) and Kapapa et al. (2024) identified critical volumetric thresholds between 31 and 38 cm^3^, or an infarct-to-posterior fossa ratio greater than 0.25, that were strongly associated with malignant edema and neurological deterioration [7,9]. Won et al. (2024), analyzing a large multicenter cohort, reported improved survival with surgical decompression in patients whose infarct volumes exceeded 35 mL [11]. Hernández-Durán et al. (2024) showed that early infarct volume reduction correlated with better functional outcomes, highlighting the importance of timely intervention [12].

In contrast, Goulin Lippi Fernandes et al. (2022) [13] found that neither surgical timing nor the degree of mass effect alone reliably predicted outcomes, reinforcing the greater prognostic utility of volumetric assessment. Additional studies (Kim 2016; Suyama 2019; Villalobos-Díaz 2022 [10,14,15]) emphasized that clinical presentation, particularly low Glasgow Coma Scale (GCS) scores and the presence of hydrocephalus, was also predictive of outcome and essential for surgical decision-making. Lucia et al. (2023) specifically found that patients with preoperative GCS scores of 12–15 experienced significant benefit from early suboccipital decompression (SDC) [8].

#### 3.2.4. Surgical Management and Timing

Suboccipital decompressive craniectomy (SDC) was consistently reported as an effective and often lifesaving, intervention for managing large cerebellar infarctions with mass effect. Kim et al. (2016) demonstrated that early [10], preemptive SDC (performed within approximately 72 h of symptom onset, before clinical deterioration) led to significantly better 12-month outcomes, with 67% of patients achieving functional independence (modified Rankin Scale score 0–2) and lower mortality compared to medically managed controls.

A systematic review by Ayling et al. (2018) [16] reported favorable outcomes across pooled data, with a mortality rate of ~20% and most survivors retaining good to moderate functional status. External ventricular drainage (EVD) was commonly employed in conjunction with SDC, particularly to manage obstructive hydrocephalus. Hernández-Durán et al. (2024) found that 86% of neurosurgical centers routinely used EVD during SDC procedures for cerebellar stroke [12]. Meta-analytic data suggested that higher EVD usage was associated with reduced mortality by Ayling et al. (2018) [16]. Larger infarcts were more likely to undergo surgical intervention, with mean infarct volumes around 53 cm^3^ in surgically treated patients versus 26 cm^3^ in those treated conservatively. Volume thresholds of ~30–31 cm^3^ were frequently cited as indicative of high risk for deterioration, warranting decompression.

Importantly, the presence of concurrent brainstem infarction was not regarded as a contraindication for surgery, although patients without brainstem involvement tended to have more favorable outcomes showed by Kim et al. (2016) [10]. Across studies, the best outcomes were consistently observed when SDC (often combined with EVD) was performed early—before irreversible brainstem compression or deep coma occurred.

#### 3.2.5. Minimally Invasive and Endoscopic Alternatives

Several studies explored less invasive surgical strategies aimed at reducing the morbidity associated with open craniectomy. Mostofi et al. (2024) compared traditional SDC with a minimally invasive endoscopic necrosectomy (MEN) technique and found similar postoperative neurological improvements in both groups [17]. The endoscopic approach offered procedural advantages, including shorter operative time and faster wound healing due to the use of a keyhole craniectomy.

Hernández-Durán et al. (2020) reported comparable outcomes using a limited osteoplastic suboccipital craniotomy with direct infarct removal (cerebellar necrosectomy) [18], achieving good functional outcomes in 76% of cases (Glasgow Outcome Scale ≥ 4) and a 30-day mortality rate of 21%. Additionally, this technique appeared to lower the risk of complications such as infection or CSF leakage by preserving the bone flap and avoiding wide dural opening (Table 2).

## 4. Discussion

SCA infarction represents a rare but clinically significant subtype of posterior circulation stroke [1,2]. Its distinct vascular supply and predilection for the upper cerebellum and rostral brainstem lead to variable and sometimes deceptive imaging findings. As highlighted in previous work, early differentiation between primary brainstem infarction and secondary compression from cerebellar edema is vital, since management and prognosis differ substantially [2,3,4,5,6]. This distinction is particularly relevant in SCA infarction, where swelling patterns frequently produce radiological appearances that do not correlate with underlying tissue viability.

The present synthesis, together with the illustrative case, emphasizes the diagnostic and therapeutic challenges of malignant SCA infarction. In the reported patient, initial CT suggested extensive brainstem involvement, raising concern for irreversible injury and poor prognosis. However, subsequent MRI demonstrated that the apparent hypodensity represented predominantly vasogenic edema rather than cytotoxic infarction—an observation that directly altered the clinical course by prompting timely decompressive surgery. This diagnostic pitfall underscores the limitations of CT in the posterior fossa, where beam-hardening artifacts and partial-volume effects can obscure subtle differences between infarction and swelling. Across multiple contemporary studies, MRI consistently demonstrated superior sensitivity to CT for detecting infratentorial ischemia, clarifying mass effect, and identifying salvageable tissue, supporting its use whenever CT and clinical findings are incongruent [5,6,7].

When synthesizing the reviewed studies, infarct volume was the parameter most consistently associated with malignant progression. Thresholds above 30–35 mL, or an infarct-to-posterior-fossa ratio exceeding 0.25, consistently predicted malignant edema and neurological decline [7,9,11]. These quantitative metrics were reproducible across heterogeneous cohorts, underscoring their utility for stratifying patients who are at risk for rapid deterioration. Early SDC, particularly in patients with preserved consciousness (GCS ≥ 12), was associated with favorable functional outcomes and reduced mortality [8,15,20]. Conversely, delayed or purely conservative management often resulted in rapid deterioration due to progressive brainstem compression. The case presented here aligns with these findings, supporting early surgical intervention once mass effect becomes evident.

Suboccipital decompressive craniectomy, with or without ventricular drainage, remains the cornerstone of therapy in space-occupying cerebellar infarction [4,7,8,21,22,23,24,25]. More recently, minimally invasive and endoscopic approaches have emerged as potential alternatives, but current evidence remains limited [11,17,20]. These techniques may reduce surgical morbidity and are promising for focal necrosis evacuation, but they do not yet replace standard decompression in cases with impending herniation or large-volume edema.

However, some authors have cautioned that preemptive decompression may expose patients to surgical risks who might otherwise have had a benign course. Reported complication rates of suboccipital decompression, including CSF leakage, wound infection, and postoperative hydrocephalus, range approximately from 10–25% in contemporary series, underscoring the need for careful selection and volumetric thresholds to avoid overtreatment [16,26]. The decision to operate therefore requires balancing the natural history of swelling, the patient’s neurological status, and the expected benefit of early versus delayed intervention, highlighting the need for individualized management.

Emerging minimally invasive approaches, including endoscopic or osteoplastic necrosectomy, offer potential advantages in selected patients by minimizing surgical trauma and promoting faster recovery [17,18,21]. However, evidence remains limited and these techniques should currently be considered complementary rather than replacements for standard SDC. Their use may be particularly justified in patients with moderate edema and localized necrosis, but not as substitutes for decompression in space-occupying lesions with threatened brainstem herniation. Future studies will need to determine which subgroups benefit most from these novel techniques and whether they can meaningfully influence long-term functional outcomes.

Notably, compared with PICA or AICA territory infarctions, SCA infarctions more often present with isolated cerebellar swelling and downward brainstem compression while less frequently showing primary brainstem ischemia on definitive imaging. This topographic distinction, also reflected in several of the included cohorts, supports analyzing SCA infarctions separately rather than pooling them with other cerebellar territories for prognostic and therapeutic decision-making [7,10,11]. Failure to distinguish these infarction patterns may lead to inaccurate outcome prediction and inappropriate extrapolation of surgical thresholds.

Based on the available evidence and the case presented, several practical steps can guide diagnostic and therapeutic decisions in suspected malignant SCA infarction. First, initial CT should be used to exclude hemorrhage, but equivocal hypodensity in the brainstem mandates urgent MRI with DWI and ADC to differentiate cytotoxic infarction from vasogenic edema. Second, early volumetric assessment (>30–35 mL or an infarct-to-posterior-fossa ratio >0.25) and evaluation of brainstem compression help identify patients at risk of malignant evolution. Third, in patients with preserved or only moderately impaired consciousness, early suboccipital decompression, with or without ventricular drainage, should be considered once space-occupying edema becomes evident. Minimally invasive approaches may be reserved for selected cases with localized necrosis but are not substitutes for decompression in mass-effect physiology. This structured sequence reflects the core diagnostic–therapeutic pathway supported by current literature.

Taken together, current evidence and the illustrative case converge on a crucial diagnostic principle: in SCA infarction, a hypodense brainstem on CT does not necessarily indicate infarction. Whenever CT suggests brainstem involvement or when clinical findings and imaging appear discordant, urgent MRI, preferably with diffusion-weighted and ADC sequences, must be performed. Only MRI can reliably distinguish between cytotoxic edema and infarction and thereby guide life-saving surgical decision-making [5,6]. As such, early multimodal imaging should be integrated into standard care pathways for suspected malignant SCA infarction to avoid preventable morbidity and mortality.

## 5. Conclusions

SCA infarction is a rare but diagnostically challenging posterior circulation stroke. Due to its anatomy, cerebellar swelling can cause downward brainstem compression that mimics primary brainstem infarction on CT, risking misdiagnosis and premature treatment withdrawal. MRI, particularly diffusion and perfusion imaging, offers critical diagnostic clarity by distinguishing true infarction from reversible edema. Volumetric and topographic assessments support timely surgical decisions, with early decompressive craniectomy, with or without ventricular drainage, remaining the mainstay of treatment. Minimally invasive and endoscopic techniques show potential but require further validation. Future studies should focus on defining standardized imaging thresholds and refining criteria for patient selection. In all cases with suspected brainstem involvement, early MRI and a structured, interdisciplinary approach are essential to improving outcomes in malignant SCA infarction.

## Figures and Tables

**Table 1:** Clinical timeline of the patient.

Time from Symptom Onset	Event/Intervention	Key Findings/Outcome
0 h	Symptom onset	Acute severe vertigo, dysphagia, dysarthria, diplopia, skew deviation, hemihypesthesia (NIHSS 7)
+2 h (admission)	Initial MRI (DWI) + TOF-angiography	Acute infarction of right cerebellum and vermis; thrombus at basilar tip; right SCA occlusion
+3 h	Intravenous thrombolysis	Standard-dose systemic thrombolysis initiated
+4 h	Digital subtraction angiography (DSA)	Basilar tip patent, right SCA occluded; mechanical thrombectomy attempted but unsuccessful
+6 h	CT scan	Expanding right cerebellar infarct with mass effect; apparent hypodensity in pons, midbrain, diencephalon → suspicious for brainstem infarction
+7 h	MRI follow-up	Brainstem infarction excluded; predominant cerebellar edema confirmed
+8 h	Neurosurgical intervention	Suboccipital decompression, ventricular drainage, and partial resection of necrotic tissue
Day 10	Extubation	Patient successfully weaned from ventilation
Day 14	Etiology work-up	Atrial septal aneurysm with patent foramen ovale (PFO) identified → referred to interventional cardiology

**Table 2:** summary of the key findings from the reviewed studies.

First Author	Year	Title (Short)	Study Type	Population	Key Findings
Ayling OGS [16]	2018	Suboccipital decompression for cerebellar infarction	Systematic review/meta-analysis	Cerebellar infarction	SDC is associated with better outcomes compared with decompressive surgery for hemispheric infarctions
Baki E [9]	2025	Predictors of malignant swelling	Retrospective cohort	Cerebellar infarction	Infarct volume >38 cm^3^ is associated with a swelling rate of >50%
Baek BH [19]	2023	SCA occlusion after thrombectomy	Retrospective cohort	SCA infarction	Attempts to recanalize remnant SCA occlusion may be unnecessary after basilar artery thrombectomy.
Goulin Lippi Fernandes E [13]	2022	Volumetric analysis and outcomes	Retrospective cohort	Cerebellar infarction	Surgical timing, including preventive surgery and mass effect of the infarct, in the posterior fossa is not predictive of the patients’ functional outcomes.
Hernández-Durán S [18]	2020	Cerebellar necrosectomy vs. decompression	Retrospective cohort	Malignant cerebellar infarction	No significant differences between mortality or functional outcomes
Hernández-Durán S [12]	2024	Surgical infarct volume reduction and outcomes	Retrospective multicenter cohort	Malignant cerebellar infarction	Early infarct volume reduction associated with better functional outcomes
Kapapa T [7]	2024	Volumetry as a criterion for decompression	Retrospective multicenter cohort	Cerebellar infarction	Volumetric cut-of >31 cm^3^ is more probable for decompression
Kim MJ [10]	2016	Preventive vs. reactive suboccipital decompression	Retrospective cohort	Cerebellar infarction	Favorable clinical outcomes including overall survival can be expected after preventive SDC in patients with a volume ratio between 0.25 and 0.33
Lindeskog D [20]	2019	Long-term outcome after decompression	Retrospective cohort	Cerebellar infarction	After SDC, half of the patients achieved a functionally acceptable level (mRS 0–3) at 12-month follow-up
Lucia K [8]	2023	Predictors of clinical outcomes	Retrospective cohort	Cerebellar infarction	Patients with space-occupying cerebellar infarction and a preoperative GCS of 12–15 significantly benefit from early SDC
Mostofi K [17]	2024	Craniectomy vs. endoscopic surgery	Retrospective cohort	Cerebellar infarction	Endoscopic vacuation of necrotic tissue is a promising alternative to decompressive craniectomy with comparable clinical outcomes.
Suyama Y [15]	2019	Significance of decompression	Retrospective cohort	Cerebellar infarction	Early DSC should be considered for treating cerebellar infarction in patients with GCS 13 or worse
Villalobos-Díaz R [14]	2022	Long-term outcomes of cerebellar strokes	Retrospective cohort	Cerebellar infarction	GCS and hydrocephalus are crucial factors in therapeutic decision-making
Won SY [11]	2024	Surgical vs. conservative treatment	Retrospective multicenter cohort	Cerebellar infarction	Surgery beneficial for infarcts >35 mL
Yoh N [21]	2023	Minimally invasive evacuation	Systematic review and case series	Spontaneous cerebellar hemorrhage	Minimally invasive evacuation is safe and effective.

## Data Availability

The original contributions presented in this study are included in the article. Further inquiries can be directed to the corresponding author.

## Supplementary Materials

The following supporting information can be downloaded at: https://www.mdpi.com/article/10.3390/jcm14228229/s1, PRISMA 2020 Checklist [27].

## Abbreviations

The following abbreviations are used in this manuscript:

ADC	Apparent Diffusion Coefficient
AI	Artificial Intelligence
AICA	Anterior Inferior Cerebellar Artery
CT	Computed Tomography
DSA	Digital Subtraction Angiography
DWI	Diffusion-Weighted Imaging
EVD	External ventricular drainage
FLAIR	Fluid-Attenuated Inversion Recovery
GCS	Glasgow Coma Scale
MEN	Minimally invasive endoscopic necrosectomy
MRI	Magnetic Resonance Imaging
NIHSS	National Institutes of Health Stroke Scale
PICA	Posterior Inferior Cerebellar Artery
PFO	Patent Foramen Ovale
PWI	Perfusion-Weighted Imaging
SANRA	Scale for the Assessment of Narrative Review Articles
SCA	Superior Cerebellar Artery
TOF	Time-of-Flight (angiography)
